# Supplementary material for: The orphan GPR50 receptor promotes constitutive TGFβ receptor signaling and protects against cancer development
Source: Nat Commun. 2018 Mar 23;9:1216. doi: 10.1038/s41467-018-03609-x (PMC5865211; doi:10.1038/s41467-018-03609-x)
Supplement: Supplementary file 1 — Supplementary Information(PDF 3080 kb) [file 41467_2018_3609_MOESM1_ESM.pdf]

Supplementary Information

"The orphan GPR50 receptor promotes constitutive TGFβ receptor signaling and protects against cancer development"

Wojciech & Ahmad et al.

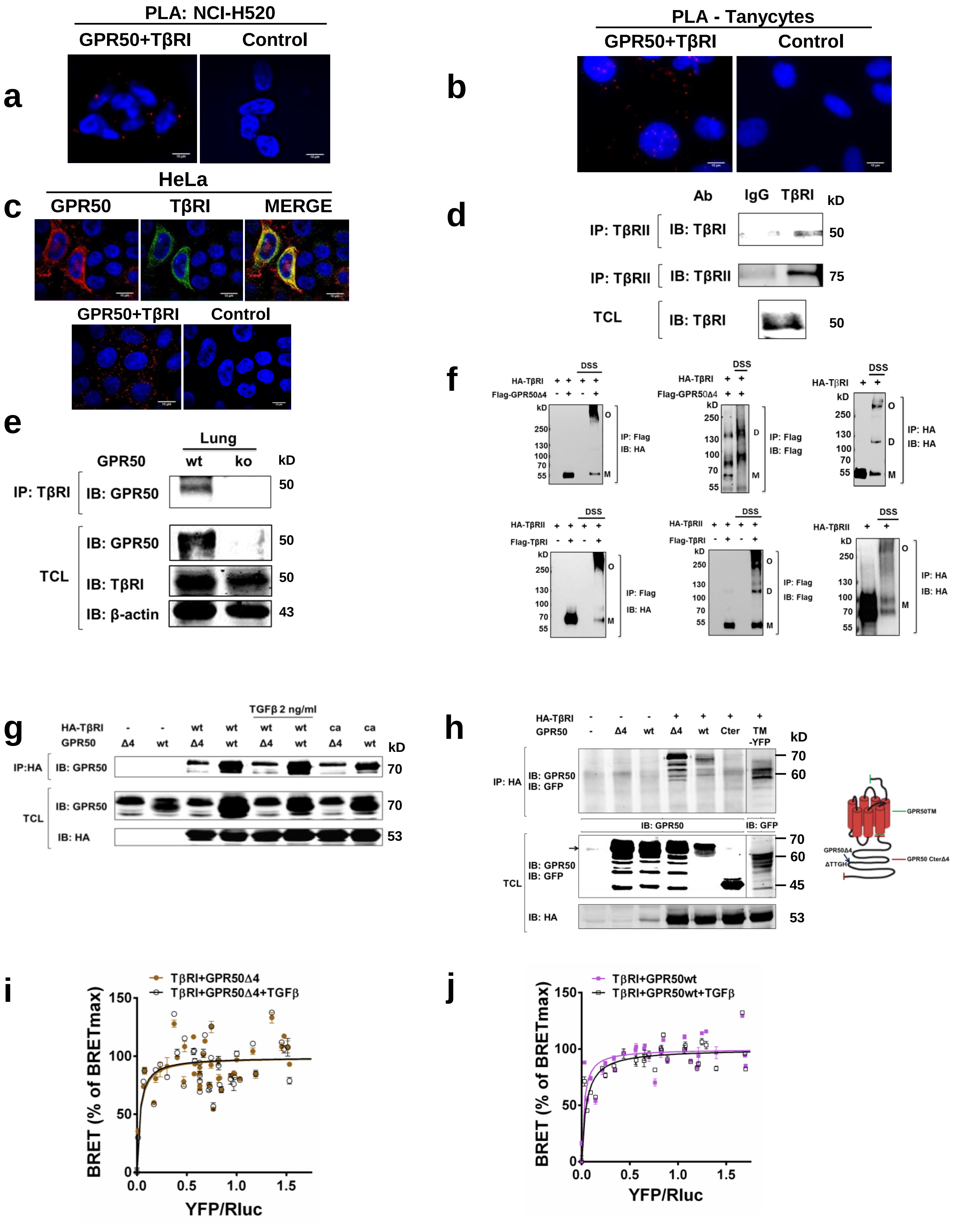

a
PLA: NCI-H520
GPR50+TβRI
Control
b
PLA - Tanycytes
GPR50+TβRI
Control
c
HeLa
GPR50
TβRI
MERGE
GPR50+TβRI
Control
d
Ab
IgG
TβRI
kD
IP: TβRII
IB: TβRI
50
IP: TβRII
IB: TβRII
75
TCL
IB: TβRI
50
e
Lung
GPR50
wt
ko
kD
IP: TβRI
IB: GPR50
50
TCL
IB: GPR50
50
IB: TβRI
50
IB: β-actin
43
f
DSS
HA-TβRI
Flag-GPR50Δ4
IP: Flag
IB: HA
IB: Flag
IP: HA
HA-TβRII
Flag-TβRI
O
D
M
g
TGFβ 2 ng/ml
HA-TβRI
GPR50
kD
IP:HA
IB: GPR50
70
TCL
IB: GPR50
70
IB: HA
53
h
HA-TβRI
GPR50
Δ4
wt
Cter
TM -YFP
kD
IP: HA
IB: GPR50
IB: GFP
70
60
TCL
45
53
GPR50TM
GPR50Δ4
ΔTTGH
GPR50 CterΔ4
i
TβRI+GPR50Δ4
TβRI+GPR50Δ4+TGFβ
BRET (% of BRETmax)
YFP/Rluc
j
TβRI+GPR50wt
TβRI+GPR50wt+TGFβ

Supplementary Figure 1. GPR50 interacts with TβRI, Related to Figure 1

(a, b) Left images visualize in situ proximity ligation assay (PLA) of the GPR50/TβRI interaction in NCI-H520 cells (a) and primary rat tanycytes (b). Right panel shows control with only one primary antibody (scale: 10µm).

(c) Upper panel shows confocal images of GPR50Δ4 and TβRI staining in co-transfected HeLa cells. Lower panel shows GPR50/TβRI interaction by PLA. Reaction without primary antibody used as negative control (scale: 10µm).

(d) NCI-H520 cells were stimulated with TGFβ (2 ng/mL; 1h) and TβRI was detected in TβRII immunoprecipitated lysate. Below are inputs (TβRII after precipitation and TβRI in lysate). Lysate incubated with IgG served as negative control.

(e) Co-immunoprecipitation of GRP50 and TβRI in lysate of lung isolated from wild type (wt) or GPR50 knock out (ko) mice. Lysate incubated with IgG served as negative control.

(f) HEK 293T cells were transfected with alone Flag-GPR50Δ4, HA-TβRI, HA-TβRII or cotransfected. Cell lysates were stimulated or not with DSS (0.5mM, 30 min, RT) and precipitation was carried out by depicted antibodies and was immunoblotted to reveal different complexes by using respective antibodies as shown. Where, monomer (M), dimer (D), oligomers (O).

(g) HEK293T cells co-transfected with GPR50Δ4 or GPR50wt and HA-TβRI or the constitutively active HA-TβRI-ca form. Stimulation with TGFβ (2ng/mL; 1h). Cell lysates were subjected to co-immunoprecipitation with anti-HA antibody and blotted against GPR50. Expression was checked in total lysates.

(h) HEK293T cells transfected with HA-TβRI and indicated GPR50 constructs. Lysates were precipitated with an anti-HA antibody and blotted against GPR50 or GFP. Total lysates were analyzed for expression. Different GPR50 constructs and the position of the Δ4 mutation is schematically illustrated on the right.

(i, j) For BRET donor saturation experiments HEK293T cells transfected with constant amount of TβRI-Rluc8 and increasing doses of GPR50Δ4-YFP (G) or GPR50wt-YFP (H). Cells were incubated with TGFβ (2 ng/mL; 15-30 min.) or PBS and BRET signals measured. Curves were normalized to BRETmax values. Saturation curves were obtained from three independent experiments performed in triplicates.

Representative results are shown for panels a-h. Similar results were obtained in at least two additional experiments.

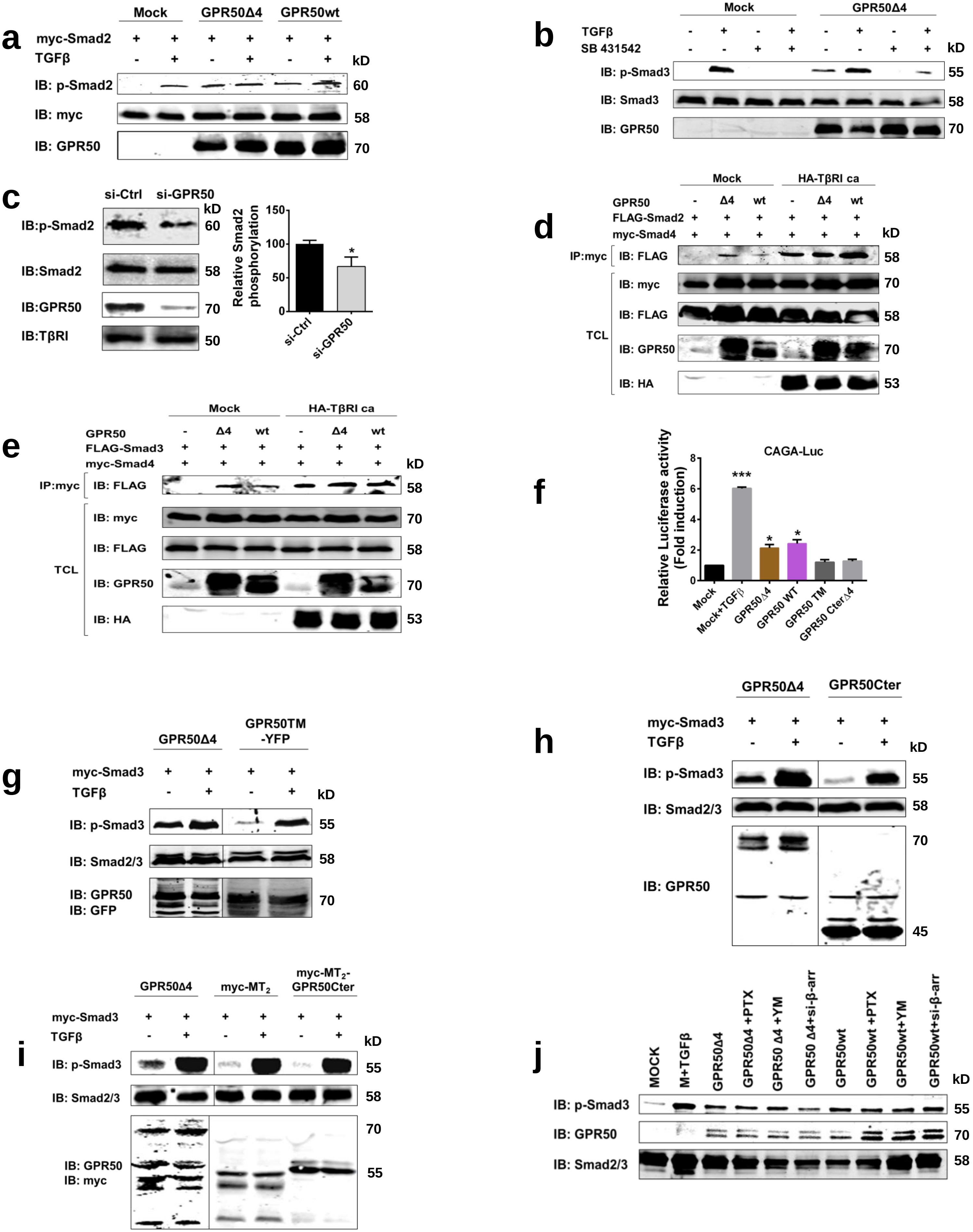
a
Mock
GPR50Δ4
GPR50wt
myc-Smad2
TGFβ
kD
IB: p-Smad2
IB: myc
IB: GPR50
60
58
70
b
Mock
GPR50Δ4
TGFβ
SB 431542
kD
IB: p-Smad3
IB: Smad3
IB: GPR50
55
58
70
c
si-Ctrl
si-GPR50
kD
IB:p-Smad2
IB:Smad2
IB:GPR50
IB:TβRI
60
58
70
50
Relative Smad2 phosphorylation
150
100
50
0
*
si-Ctrl
si-GPR50
d
Mock
HA-TβRI ca
GPR50
Δ4
wt
FLAG-Smad2
myc-Smad4
kD
IP:myc
IB: FLAG
TCL
IB: myc
IB: FLAG
IB: GPR50
IB: HA
58
70
58
70
53
e
Mock
HA-TβRI ca
GPR50
Δ4
wt
FLAG-Smad3
myc-Smad4
kD
IP:myc
IB: FLAG
TCL
IB: myc
IB: FLAG
IB: GPR50
IB: HA
58
70
58
70
53
f
CAGA-Luc
Relative Luciferase activity (Fold induction)
8
6
4
2
0
***
*
*
Mock
Mock+TGFβ
GPR50Δ4
GPR50 WT
GPR50 TM
GPR50 CterΔ4
g
GPR50Δ4
GPR50TM -YFP
myc-Smad3
TGFβ
kD
IB: p-Smad3
IB: Smad2/3
IB: GPR50
IB: GFP
55
58
70
h
GPR50Δ4
GPR50Cter
myc-Smad3
TGFβ
kD
IB: p-Smad3
IB: Smad2/3
IB: GPR50
55
58
70
45
i
GPR50Δ4
myc-MT2
myc-MT2-GPR50Cter
myc-Smad3
TGFβ
kD
IB: p-Smad3
IB: Smad2/3
IB: GPR50
IB: myc
55
58
70
55
j
MOCK
M+TGFβ
GPR50Δ4
GPR50Δ4 +PTX
GPR50 Δ4 +YM
GPR50 Δ4+si-β-arr
GPR50wt
GPR50wt +PTX
GPR50wt+YM
GPR50wt+si-β-arr
kD
IB: p-Smad3
IB: GPR50
IB: Smad2/3
55
70
58

Supplementary Figure 2. GPR50 promotes TβRI signaling, Related to Figure 2

(a) HEK293T cells were transfected as described in Figure 2a. To determine Smad2 phosphorylation myc-Smad2 was enriched by immunoprecipitation with an anti-myc antibody and blotted for p-Smad2.
(b) HEK293T cells were transfected and prepared as described in Figure 2a but pretreated overnight with 10 µM of SB 431542 (TβRI-kinase inhibitor).
(c) p-Smad2 expression in NCI-H520 cells following the silencing of GPR50 by si-RNA. Control si-RNA (si-Ctrl) served as control. Densitometric analysis of 3 independent experiments (Mean± s.e.m., n=3 independent experiments, *$p<0.05$ two-tailed unpaired Student's t-test).
(d, e) To monitor Smad2/4 and Smad3/4 complex formation, HEK293 cells were transfected with corresponding myc or FLAG-tagged constructs in the absence or presence of GPR50Δ4 and GPR50wt. The constitutively active TβRI-ca form served as positive control. Smad4 was precipitated with an anti-FLAG antibody and bound Smad2 or Smad3 revealed by western blot. Lysates were used for total expression.
(f) To asses TGFβ-dependent transcription of genes, HeLa cells were transfected with a Firefly-Luciferase-coupled CAGA reporter gene construct and Renilla Luciferase for normalization. The indicated plasmid plasmids were transfected; empty (Mock ± TGFβ; 0.5ng/mL, 8h), GPR50Δ4 and GPR50wt (Mean ± s.e.m., n=3 independent experiments, * $p<0.05$, *** $p<0.001$ one-way ANOVA with Dunnett's post-hoc test).
(g, h) HEK293T cells expressing GPR50Δ4 (g, h), GPR50TM-YFP (g) or GPR50Cter (h) were starved overnight and stimulated with TGFβ (2ng/mL; 1h) and p-Smad3 levels determined in cell lysates. GPR50 expression was revealed with anti-GPR50 or anti-GFP (GPR50Cter) antibodies.
(i) HEK293T cells expressing GPR50Δ4, myc-MT2 melatonin receptor or the MT2 -GPR50Cter chimera were starved overnight and stimulated with TGFβ (2ng/mL; 1h) and p-Smad3 levels determined in cell lysates. Expression of transfected proteins was confirmed in cell lysates. Representative results are shown for all the panels. Similar results were obtained in at least two additional experiments.
(j) HEK 293T cells were transfected with indicated plasmid and treated with TGF-β (2ng/mL; 1h), Gi inhibitor (PTX, 10ng/mL; O/N), Gq inhibitor (YM-254890;100nM-1h) and si-RNA against β-arrestin and blotted for p-Smad3.
Representative results are shown for panels a-e and g-j. Similar results were obtained in at least two additional experiments.

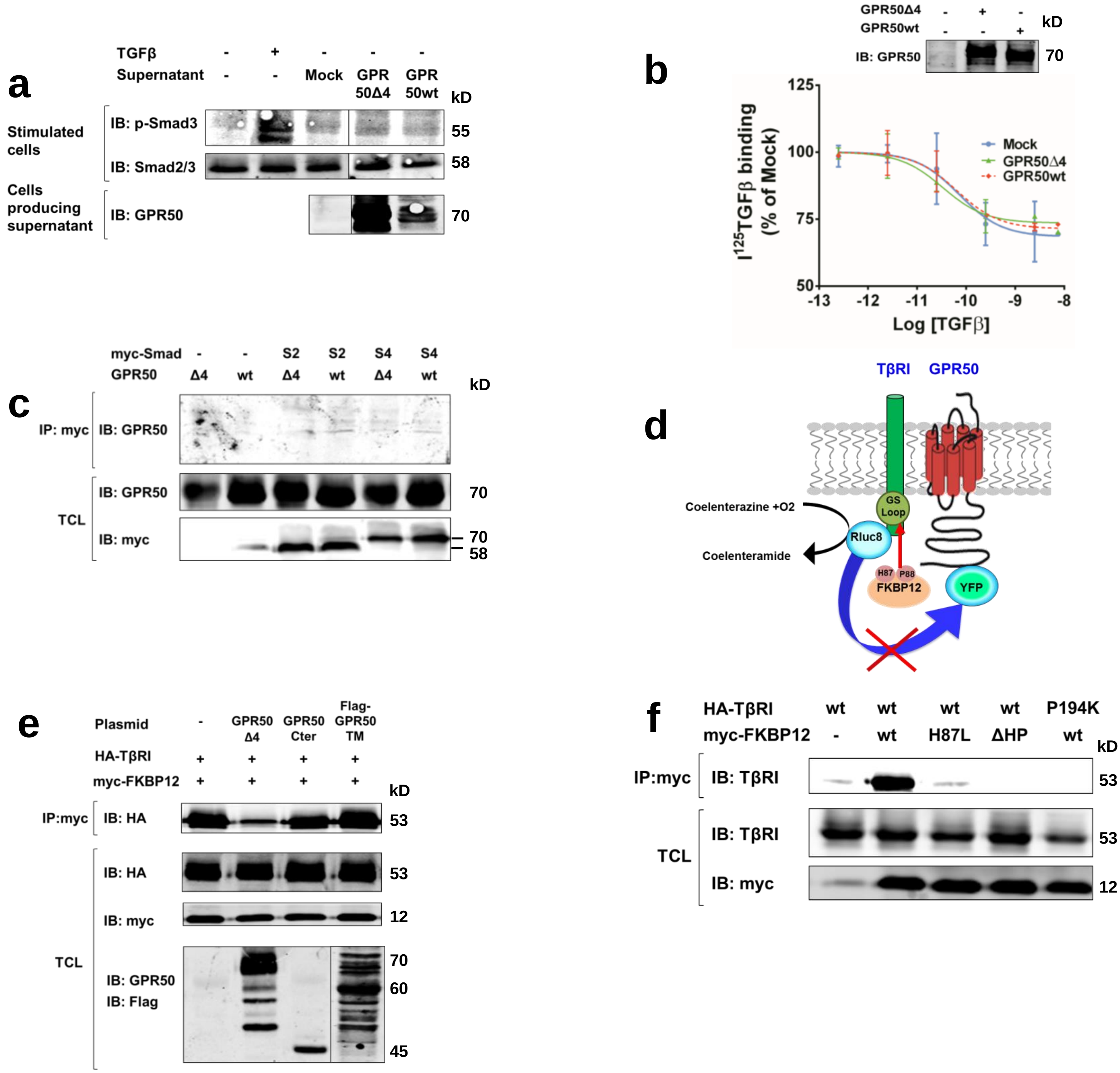

a
TGFβ - + - - -
Supernatant - - Mock GPR 50Δ4 GPR 50wt
kD
Stimulated cells
IB: p-Smad3 55
IB: Smad2/3 58
Cells producing supernatant
IB: GPR50 70
b
GPR50Δ4 - + -
GPR50wt - - +
kD
IB: GPR50 70
I125TGFβ binding (% of Mock)
Log [TGFβ]
Mock
GPR50Δ4
GPR50wt
125
100
75
50
-13 -12 -11 -10 -9 -8
c
myc-Smad - - S2 S2 S4 S4
GPR50 Δ4 wt Δ4 wt Δ4 wt
kD
IP: myc IB: GPR50
TCL
IB: GPR50 70
IB: myc 70 58
d
TβRI GPR50
GS Loop
Coelenterazine +O2
Rluc8
Coelenteramide
H87 P88
FKBP12
YFP
e
Plasmid - GPR50 Δ4 GPR50 Cter Flag-GPR50 TM
HA-TβRI + + + +
myc-FKBP12 + + + +
kD
IP:myc IB: HA 53
TCL
IB: HA 53
IB: myc 12
IB: GPR50
IB: Flag
70
60
45
f
HA-TβRI wt wt wt wt P194K
myc-FKBP12 - wt H87L ΔHP wt
kD
IP:myc IB: TβRI 53
TCL
IB: TβRI 53
IB: myc 12

Supplementary Figure 3. GPR50 interferes with FKBP12 binding to TβRI, Related to Figure 3

(a) To determine the capacity of GPR50 to promote TGFβ secretion in HEK293T cells, naive HEK293T cells were starved and treated for one hour with the supernatant of HEK293T expressing GPR50Δ4 or GPR50wt. Stimulation of the naive HEK293T cells with 2ng/mL of TGFβ served as positive control. p-Smad3 and total Smad3 levels were determined in stimulated HEK293T cells. Cells producing supernatant were checked for GPR50 expression.
(b) Competition of 125I-TGFβ binding by increasing doses of TGFβ in intact MDA-MB-231 cells expressing or not either GPR50Δ4 or GPR50wt. GPR50 expression was verified by western blot. Data represent the mean ± s.e.m. of three independent experiments performed in triplicates.
(c) Co-immunoprecipitation was performed with HEK293T cells co-transfected with myc-Smad2 or myc-Smad4 and either GPR50Δ4 or GPR50wt. Lysates were incubated with an anti-myc antibody to precipitate Smad proteins (S2, Smad2; S4, Smad4). Precipitates were blotted against GPR50 and total lysates were analyzed with an anti-myc and an anti-GPR50 antibody for expression.
(d) Schematic diagram depicting the BRET strategy used in Figure 3d to study the competition between FKBP12 and GPR50 for TβRI binding.
(e) To address the specificity of GPR50 for decreasing the interaction between TβRI and FKBP12, HEK293T cells were transfected with HA-TβRI and myc-FKBP12 and either GPR50Δ4, GPR50CterΔ4, GPR50TM. Experiments were performed according to the Figure 3a and protein expression was revealed by corresponding antibodies as indicated in the figure.
(f) HEK293T cells were co-transfected with HA-TβRI or the HA-TβRI-P194K mutant and the indicated FKBP12 constructs. Co-immunoprecipitation was performed as in Figure 3g.
Representative results are shown for panels a, c, e, f. Similar results were obtained in at least two additional experiments.

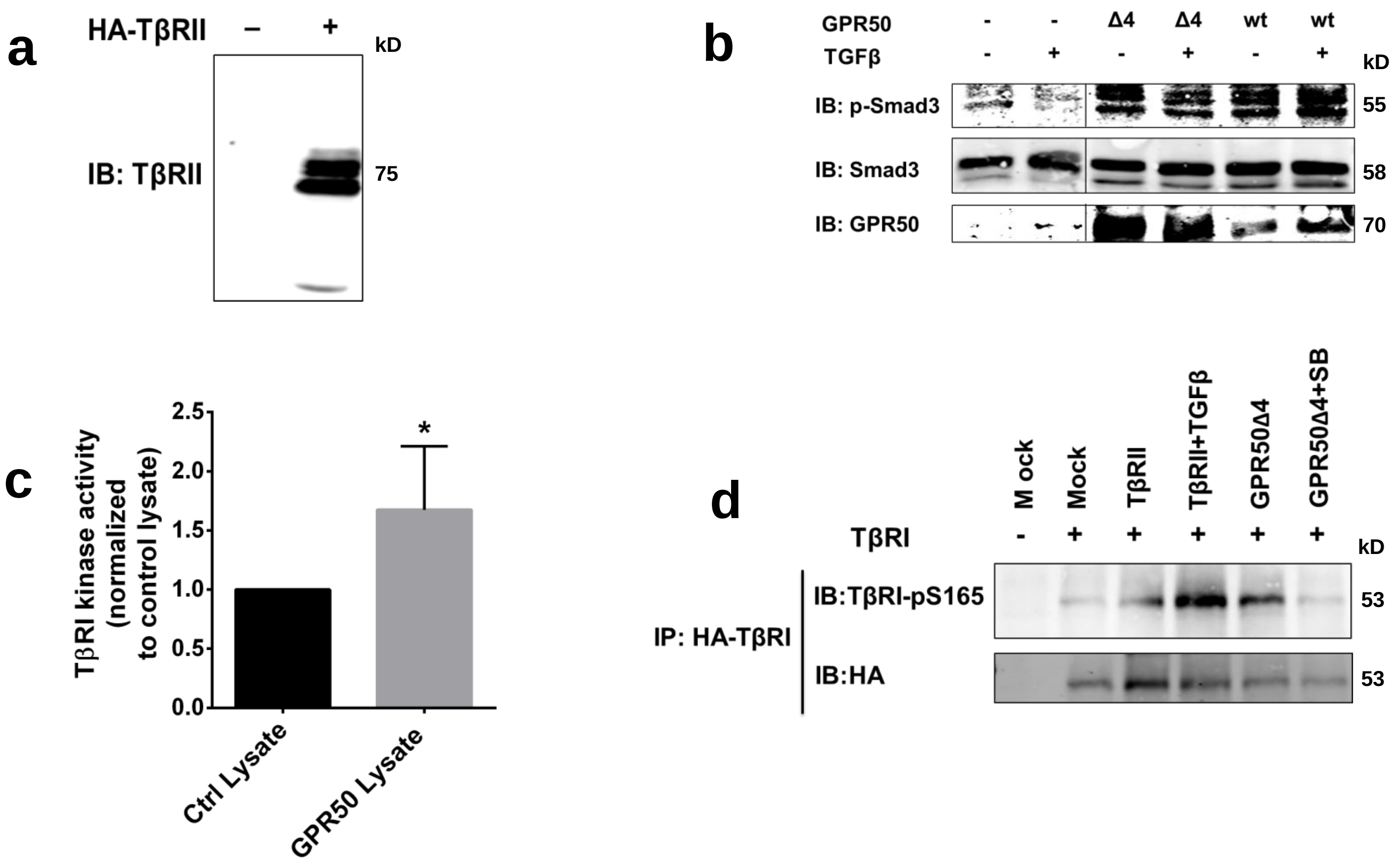

Supplementary Figure 4. GPR50 phosphorylates TβRI in the absence of TβRII, Related to Figure 4

(a) SNU638 cells expressing HA-TβRII or not were lysed and TβRII protein expression detected by western blot with anti-TβRII antibody.
(b) SNU638 cells were either mock transfected or with GPR50Δ4 or GPR50wt expression vectors and stimulated for one hour with 2 ng/mL of TGFβ. Lysates were immunoblotted for p-Smad3 and total Smad3 and expression of GPR50.
(c) GPR50Δ4 was precipitated from HEK293T cell lysates and incubated with purified TβRI kinase. Mock-transfected HEK293T cell lysates served as negative control. Kinase activity was measured according to manufacturer's instructions (Promega). (Mean± s.e.m., n=5 independent experiments, * $p<0.05$, two-tailed unpaired Student's t-test).
(d) SNU638 cells transfected either with alone TβRI, TβRII, or both with TGFβ stimulation (0.5ng/mL; 1h) or with GPR50Δ4 with and without SB 431542 at 10 µM. Total TβRI was immunoprecipitated from lysate and immunoblotted for phosphorylated TβRI (antiTβRI-pS165 antibody). Below lane shows total precipitated TβRI in lysate.
Representative results are shown for panel b. Similar results were obtained in at least two additional experiments.

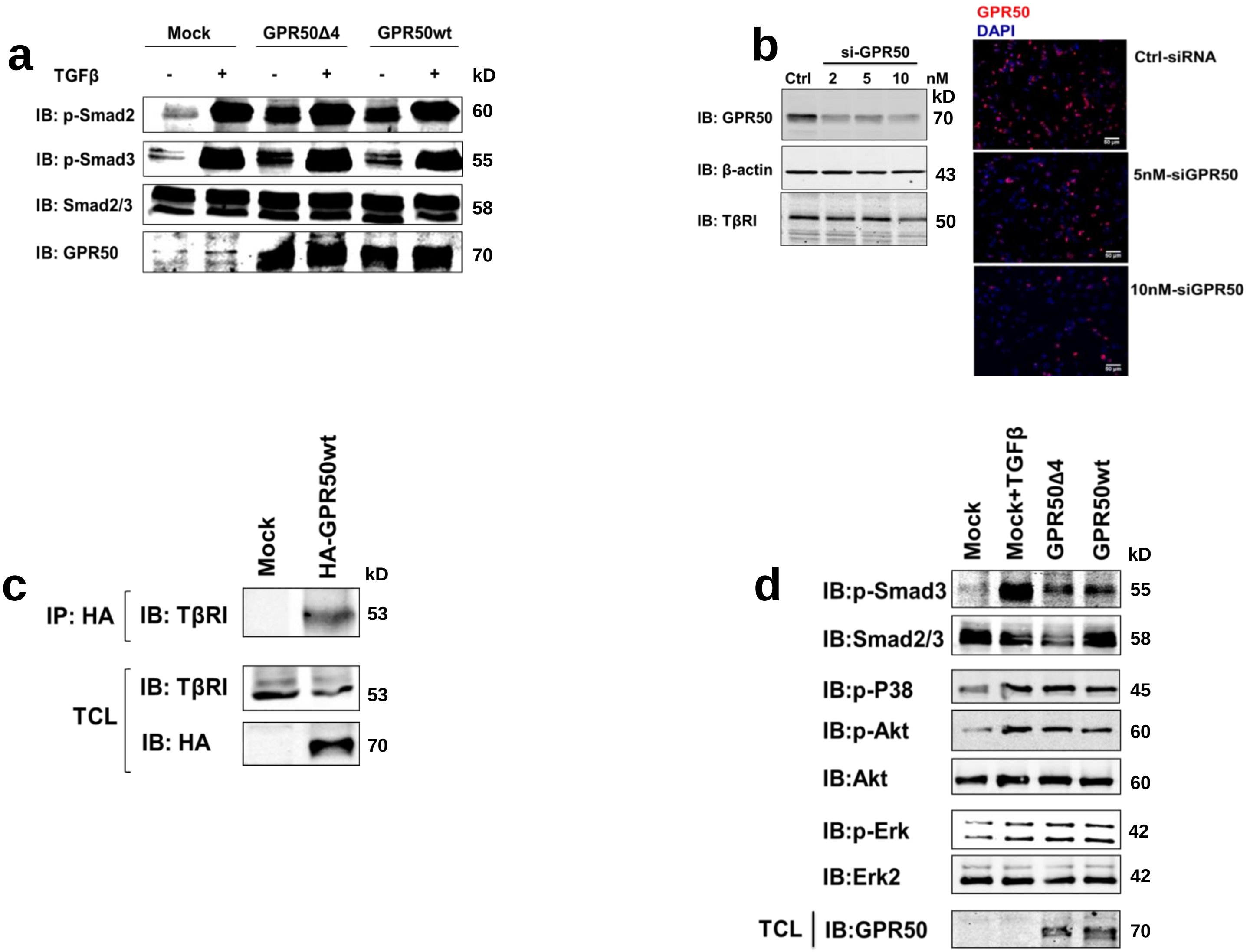

Supplementary Figure 5. GPR50 mimics TGFβ mediated cellular responses, Related to Figure 5

(a) MDA-MB-231 cells expressing GPR50Δ4 or GPR50wt were starved overnight and stimulated or not with 2 ng/mL TGFβ for one hour. The p-Smad2 and p-Smad3 levels and total expression of Smad2/3 and GPR50 were determined by western blot in cell lysates.
(b) GPR50 expression in human lung carcinoma cells (NCI-H520) treated with the indicated concentration of si-RNA against GPR50 (si-GPR50) or 10 nM of control si-RNA (Ctrl) monitored either in cell lysates by western blot (left panel) or immunostaining (right panel) (scale: 50μm). TβRI and β-actin levels were determined to control the loading.
(c) Immunoblot shows Co-immunoprecipitation of GPR50 (transfected) and TβRI (endogenous) in 4T1 cells. 4T1 cells were transfected with empty vector (Mock), and HA-GPR50wt. GPR50 was precipitated with anti-HA and blotted for TβRI by anti-TβRI. Inputs were shown in total lysate for both proteins.
(d) Immunoblot shows different p-Smad3 and non canonical phosphoproteins (p-ERK, p-Akt, p-p38) in 4T1 cell lysate following stimulation with TGF-β (2ng/mL; 1h). Where, empty vector (Mock) and GPR50 were transiently transfected.

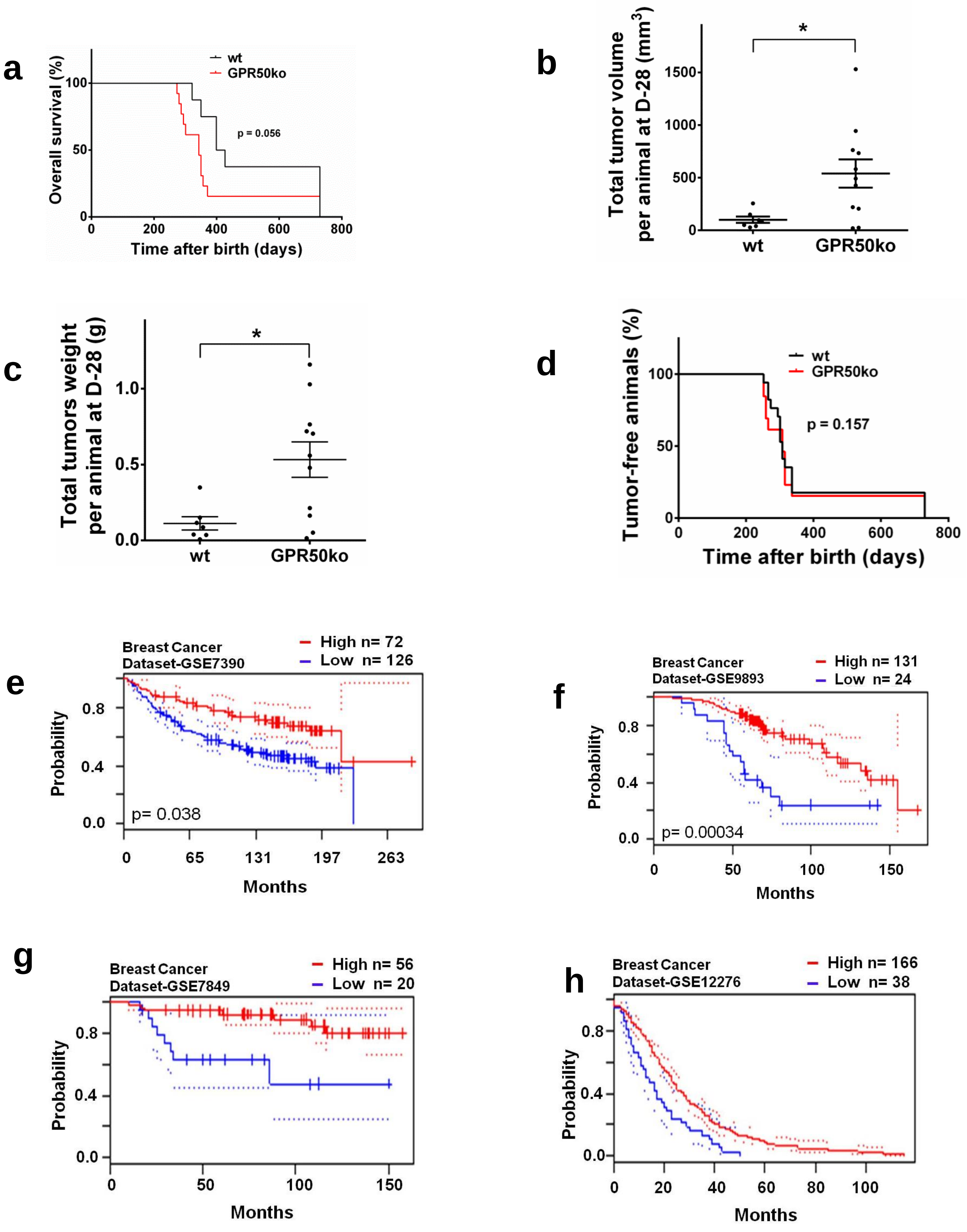

a
wt
GPR50ko
Overall survival (%)
p = 0.056
Time after birth (days)
b
Total tumor volume per animal at D-28 (mm3)
wt
GPR50ko
c
Total tumors weight per animal at D-28 (g)
wt
GPR50ko
d
Tumor-free animals (%)
wt
GPR50ko
p = 0.157
Time after birth (days)
e
Breast Cancer Dataset-GSE7390
High n= 72
Low n= 126
Probability
p= 0.038
Months
f
Breast Cancer Dataset-GSE9893
High n= 131
Low n= 24
Probability
p= 0.00034
Months
g
Breast Cancer Dataset-GSE7849
High n= 56
Low n= 20
Probability
Months
h
Breast Cancer Dataset-GSE12276
High n= 166
Low n= 38
Probability
Months

Supplementary Figure 6. GPR50 is protective against breast cancer, Related to Figure 6 and supplementary tables 1-6

(a) Overall survival curve of MMTV-Neu-wt and MMTV-Neu-GPR50ko mice where death is reported from birth onwards (Mean± s.e.m., WT n=8, ko n=13, Kaplan Meier survival curve with log-rank Mantel-Cox test, p=0.056).
(b, c) Graphs showing total tumor volume (b) and total tumor weight (c;) in MMTV-Neu-wt and MMTV-Neu-GPR50ko mice at day 28 after tumor onset (average survival day of MMTV-Neu-GPR50ko mice) (Mean± s.e.m., WT n=9, ko n=11, unpaired two tailed t-test *p<0.05) .
(d) Tumor onset curve showing tumor free animals in percentage where death is reported from birth (Mean± s.e.m., WT n=17, ko n=13 Kaplan Meier survival curve with log-rank Mantel-Cox test, p=0.157).
(e,f,g,h) Low expression of GPR50 associated with poor prognosis. Kaplan Meier survival curves showing prognosis following GPR50 under/overexpression in human breast cancer database obtained from Prognoscan.org.

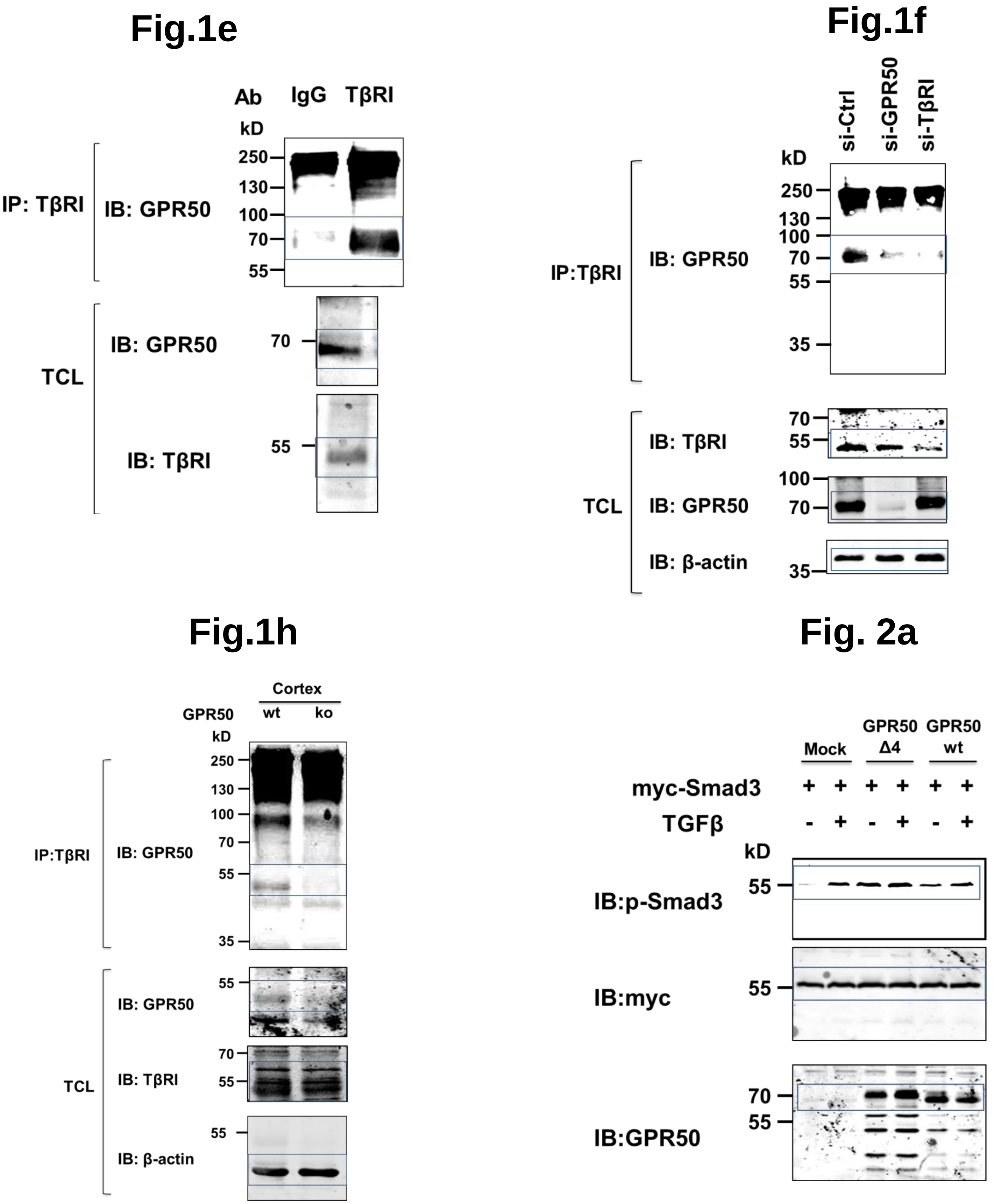

Supplementary Figure 7. Uncropped blots corresponding to the labelled figures in the manuscript

Uncropped blots showing the cropped area in blue box for figure 1e, 1f, 1h and figure 2a.

Fig. 2b

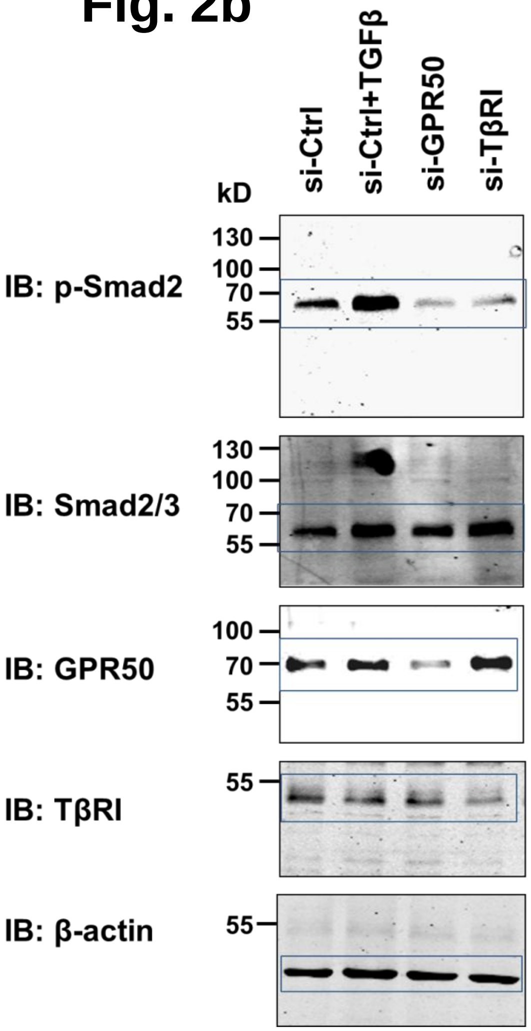

Fig. 2c

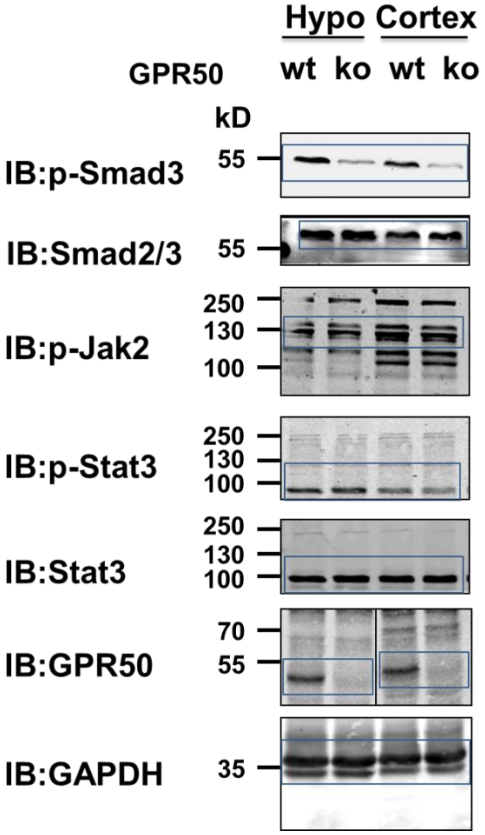

Supplementary Figure 8. Uncropped blots corresponding to the labelled figures in the manuscript

Uncropped blots showing the cropped area in blue box for figure 2b and 2c.

Fig. 2d

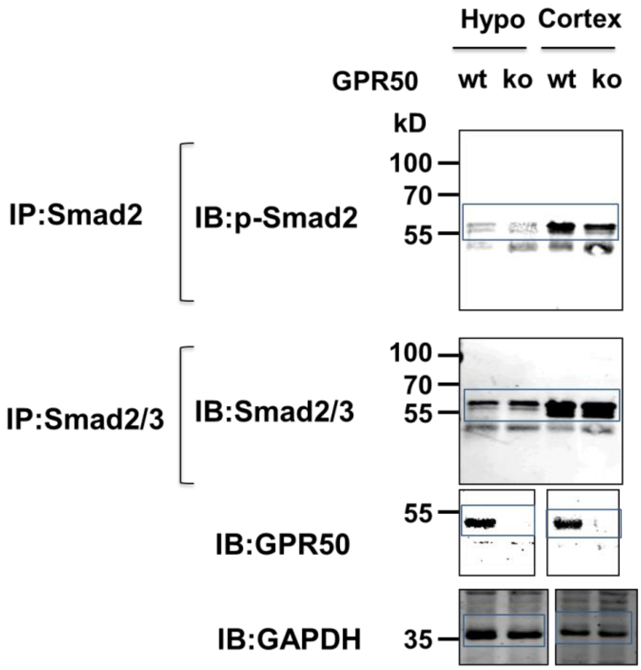

Fig. 2f

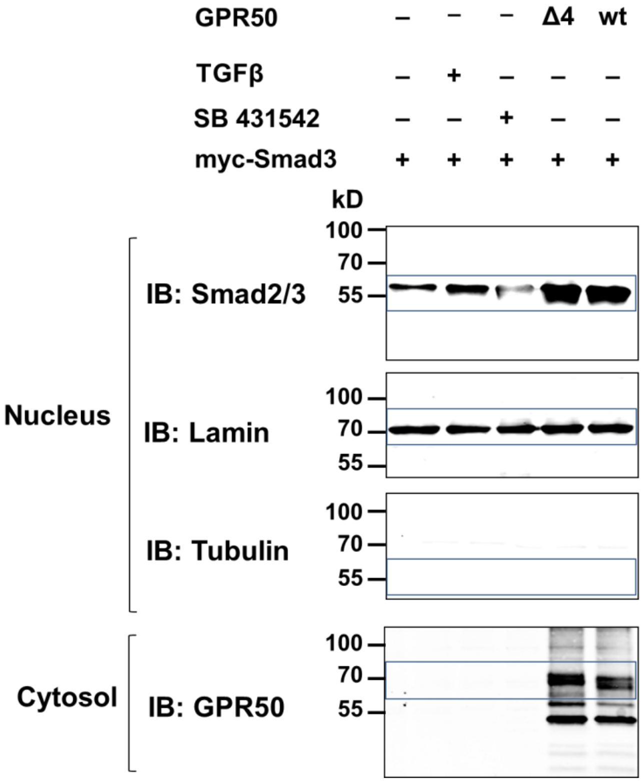

Fig. 2h

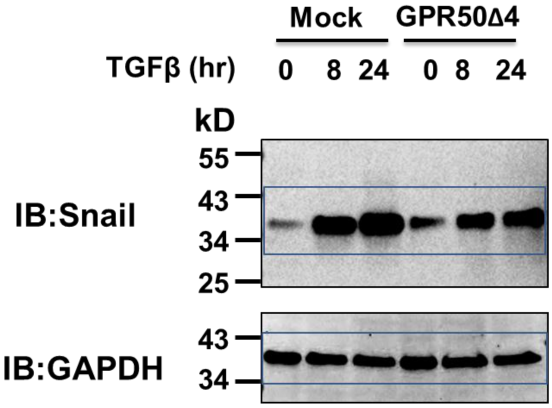

Supplementary Figure 9. Uncropped blots corresponding to the labelled figures in the manuscript

Uncropped blots showing the cropped area in blue box for figure 2d , 2f and figure 2h.

Fig. 3a

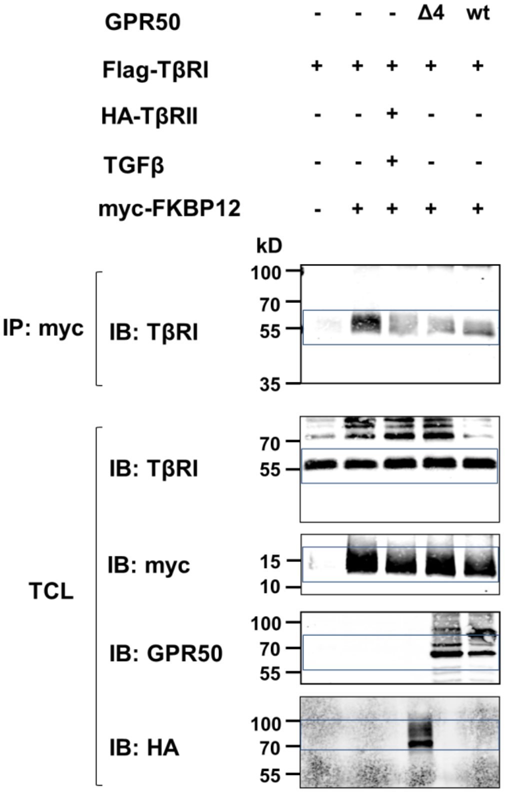

Fig. 3b

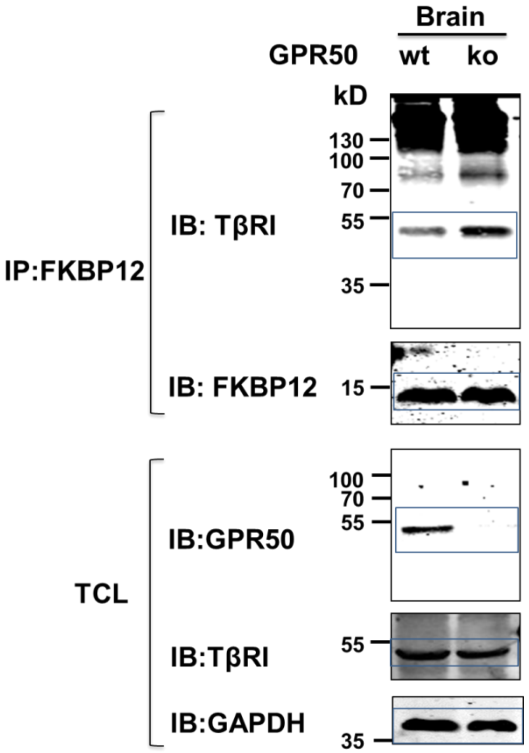

Fig. 3c

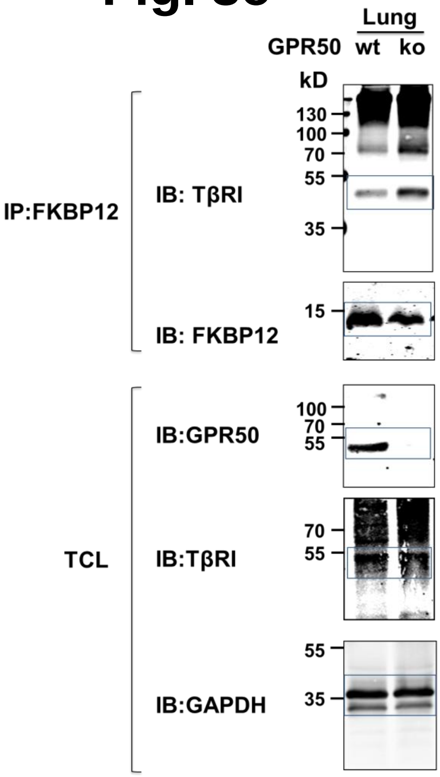

Fig. 3e

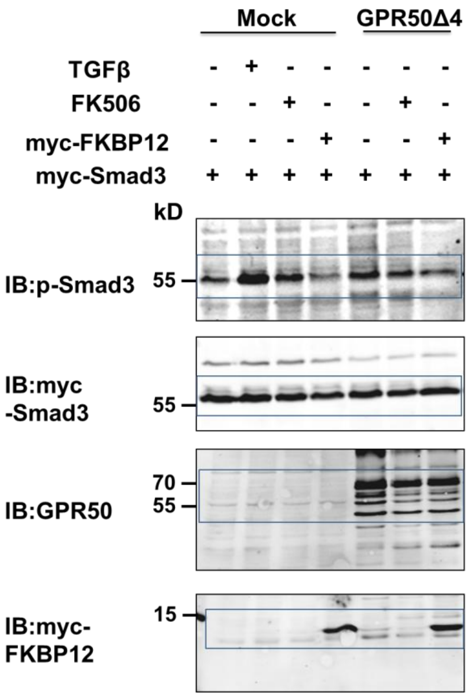

Supplementary Figure 10. Uncropped blots corresponding to the labelled figures in the manuscript

Uncropped blots showing the cropped area in blue box for figure 3a , 3b, 3c and figure 3e.

Fig. 3g

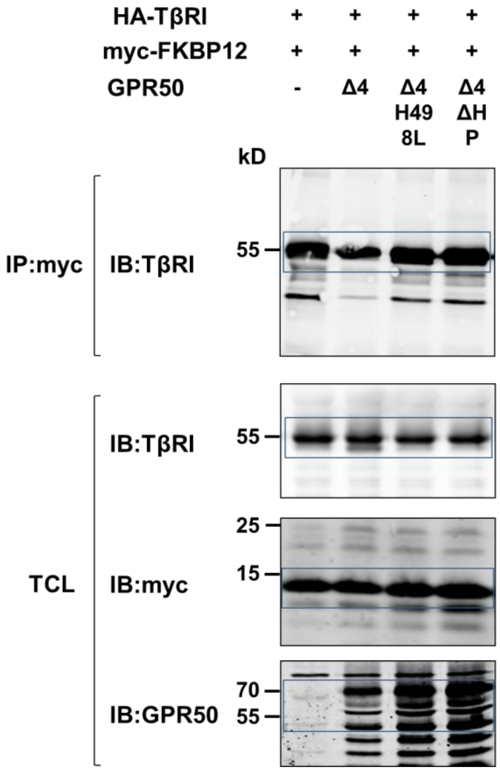

Fig. 4a

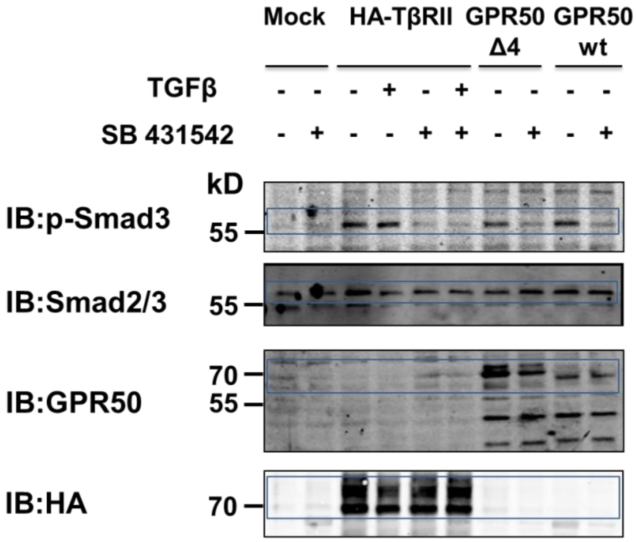

Fig. 4c

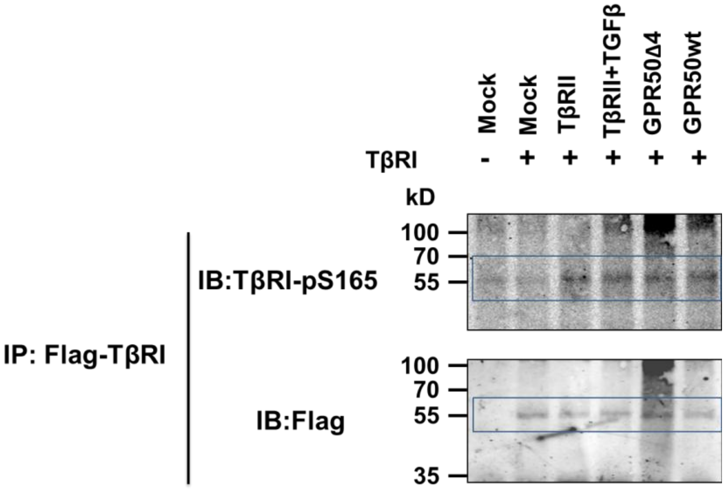

Fig. 4d

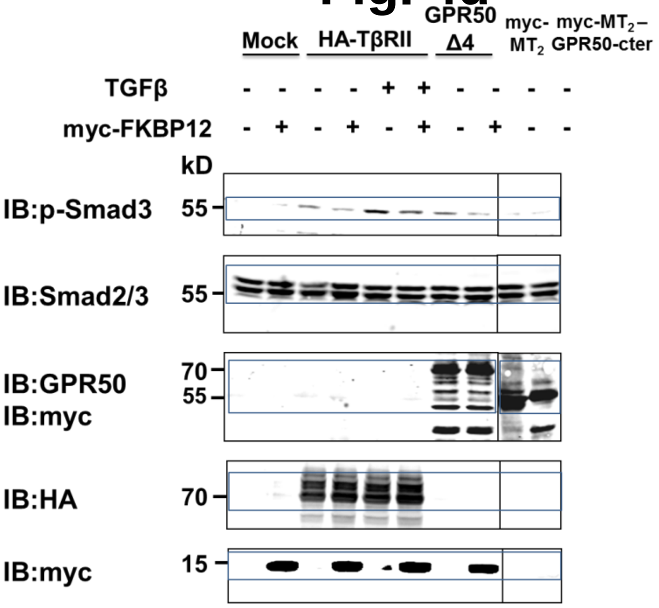

Supplementary Figure 11. Uncropped blots corresponding to the labelled figures in the manuscript

Uncropped blots showing the cropped area in blue box for figure 3g , 4a, 4c and figure 4d.

Suppl Fig.1d

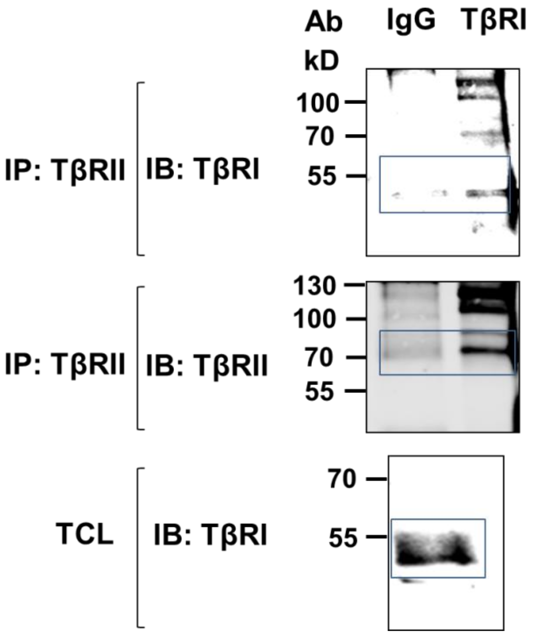

Suppl Fig. 2g

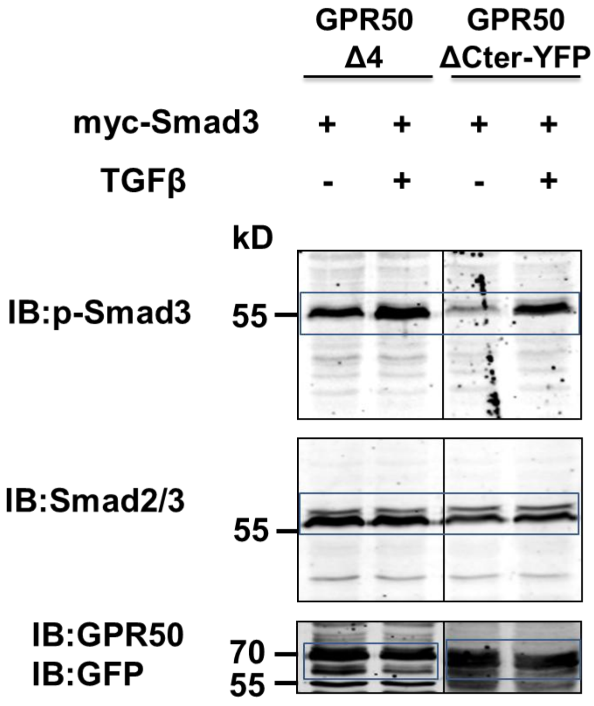

Suppl Fig. 2h

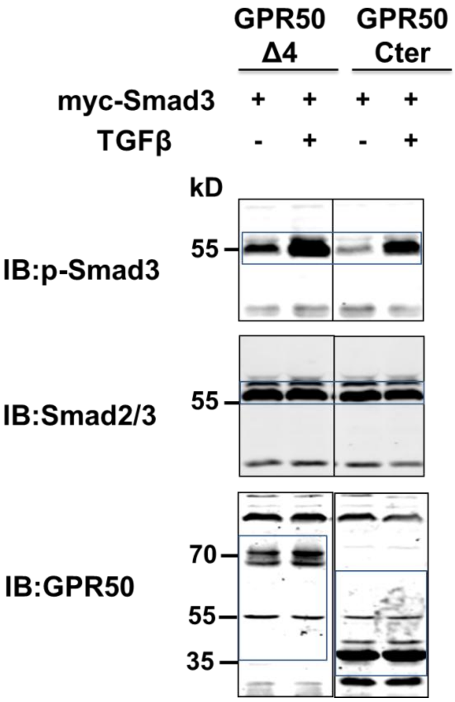

Suppl Fig. 3f

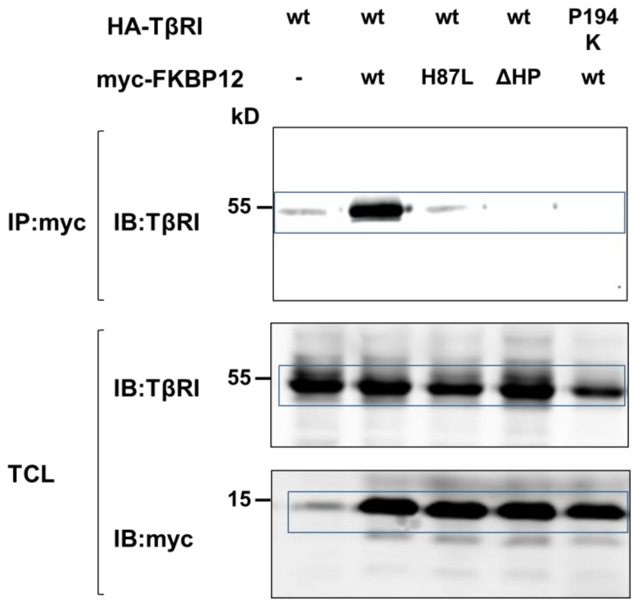

Supplementary Figure 12. Uncropped blots corresponding to the labelled figures in the manuscript

Uncropped blots showing the cropped area in blue box for supplementary figure 1d , 2g, 2h and supplementary figure 3f.

Suppl Fig. 4d

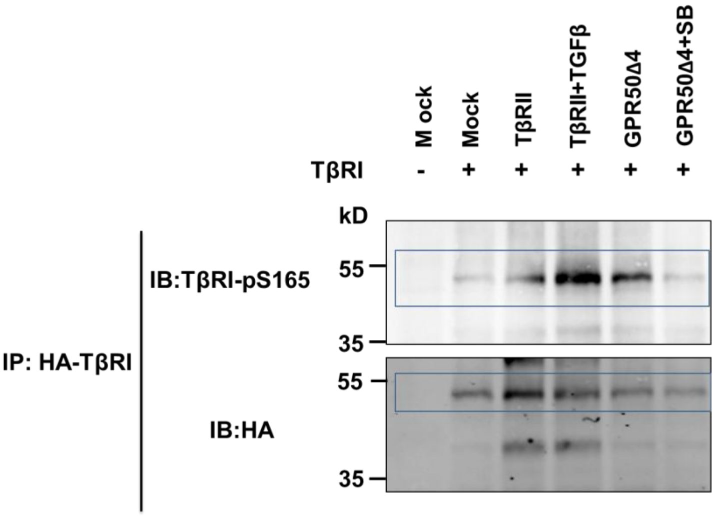

Suppl Fig. 5 d

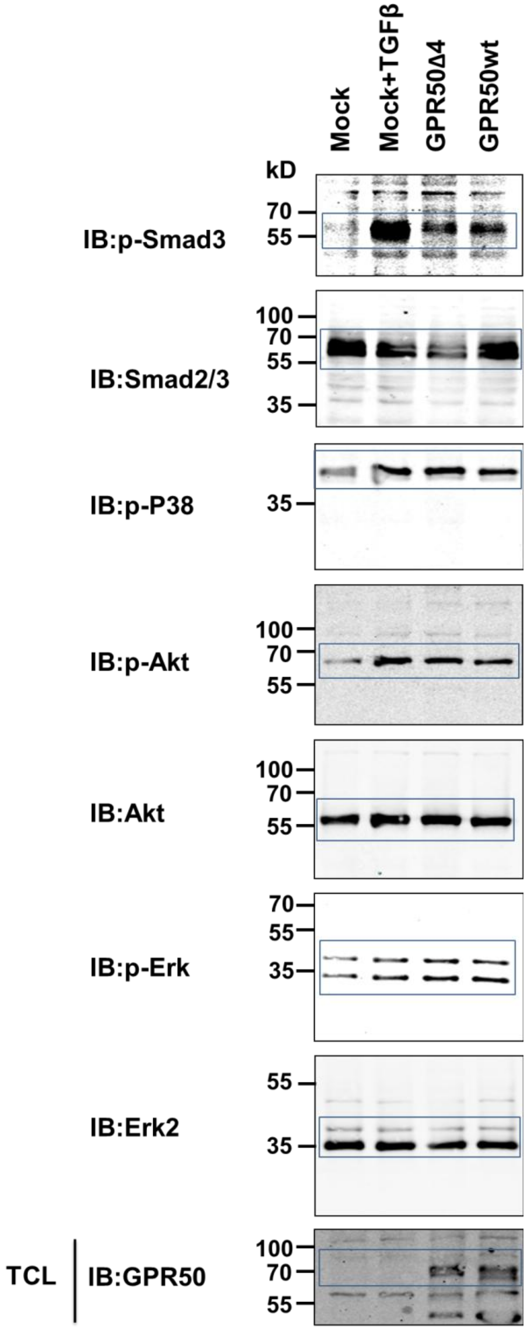

Supplementary Figure 13. Uncropped blots corresponding to the labelled figures in the manuscript

Uncropped blots showing the cropped area in blue box for supplementary figures 4d supplementary figure 5d.

Tumor Site	Oncomine Reference	Comparison	Array reporter	Fold change	P value	T test value
Kidney	Jones Renal	Oncocytoma vs Normal	208311_at	-2.32	2.04 E-13	-17
		Papillary Cell Carcinoma vs Normal	208311_at	-2.10	2.76 E-18	-20
Pancreas	Buchholz Pancreas	Ductal Adenocarcinoma vs Normal	NM_ 004224	-2.06	1.95E-4	-5.01
Breast	Radvanyi Breast	Invasive Lobular Carcinoma vs Normal	U52219	-5.07	0.04	-2.09
		Invasive Ductal Carcinoma vs Normal	U52219	-2.245	0.04	-1.95
Lung	Bhattacharjee Lung	Lung Carcinoid Tumor vs Normal	31406_at	-4.37	3.92E-5	-4.94
		Small Cell Lung Carcinoma vs Normal	31406_at	-3.95	3.08E-4	-4.04
		Lung Adenocarcinoma vs Normal	31406_at	-2.140	0.009	-2.62
Gastric	DErrico Gastric	Mixed Adenocarcinoma vs Normal	208311_at	-1.868	1.78E-4	-4.544
		Intestinal type Adenocarcinoma vs Normal	208311_at	-1.527	0.002	-2,95

Supplementary Table 1. Underexpression of GPR50 in Different Human Cancers, Related to Figure 6
Analysis of cancer vs normal tissue with the Oncomine data base considering underexpression of GPR50. Cases with significant p-value (<0.05) and 2-5 fold changes of expression are shown.

Tumour Site	Oncomine Reference	Comparison	Array reporter	Fold change	P value	T test value
Skin	Nindl Skin	Actinic (Solar) Keratosis vs Normal	208311_at	2.36	0.132	1.32
		Skin Squamous Cell Carcinoma vs Normal	208311_at	1.51	0.096	1.41
	Riker Melanoma	Skin Squamous Cell Carcinoma vs Normal	208311_at	1.81	0.02	2.29
		Cutaneous Melanoma vs Normal	208311_at	1.24	0.17	0.98
		Skin Basal Cell Carcinoma vs Normal	208311_at	-1.08	0.66	-0.45
Blood	Durig Leukemia	T-Cell Prolymphocytic Leukemia vs Normal	208311_at	1.77	0.01	2.38

Supplementary Table 2. Overexpression of GPR50 in Different Human Cancers, Related to Figure 6
Analysis of cancer vs normal tissue with the Oncomine data base considering overexpression of GPR50. Only skin and blood cancer cases come up which had less than 2 fold changes in expression and non-significant p-values (>0.05) as compared to normal tissue.

Tumor Site	Oncomine Reference	Comparison	Array reporter	Fold change	P value	T test value
Breast	TCGA Breast 2	Invasive Ductal Breast Carcinoma vs Normal	23-150098152	-1.06	6.38E-10	-6.13
		Mixed Lobular and Ductal Breast Carcinoma vs. Normal	23-150098152	-1.12	0.021	-2.42
		Invasive Lobular Breast Carcinoma vs. Normal	23-150098152	-1.05	0.026	-1.98
Breast	Curtis Breast	Ductal Breast Carcinoma in Situ vs. Normal	ILMN_1694483	-1.05	0.014	-2.4
		Medullary Breast Carcinoma vs. Normal	ILMN_1694483	-1.03	0.03	-1.91
		Breast Carcinoma vs. Normal	ILMN_1694483	-1.04	0.03	-1.85
		Invasive Ductal and Invasive Lobular Breast Carcinoma vs. Normal	ILMN_1694483	-1.02	0.05	-1.58

Supplementary Table3. Underexpression of GPR50 in Breast cancers (ER negative), Related to Figure 6
Analysis of cancer vs normal tissue with the Oncomine data base considering underexpression of GPR50 in ER-negative breast cancer. All cancer subtypes with significant P values observed.

Tumor Site	Oncomine Reference	Comparison	Array reporter	Fold change	P value	T test value
Breast	TCGA Breast	Invasive Ductal Breast Carcinoma vs. Normal	A_23_P159721	1.1	9.0E-4	3.24
Breast	Richardson Breast 2	Ductal Breast Carcinoma vs. Normal	208311_at	1.2	0.02	2.3

Supplementary Table 4. Overexpression of GPR50 in Breast Cancers (ER negative), Related to Figure 6
Analysis of cancer vs normal tissue with the Oncomine data base considering overexpression of GPR50 in ER-negative breast cancer. Only two cancer subtypes observed.

Tumor Site	Oncomine Reference	Comparison	Array reporter	Fold change	P value	T test value
Breast	TCGA Breast 2	Invasive Ductal Breast Carcinoma	23-150098152	-1.06	6.38E-10	-6.13
		Mixed Lobular and Ductal Breast Carcinoma vs. Normal	23-150098152	-1.128	0.021	-2.42
		Invasive Lobular Breast Carcinoma vs. Normal	23-150098152	-1.05	0.026	-1.98
		Invasive Papillary Breast Carcinoma vs. Normal	23-150098152	-1.01	0.05	-2.71
Breast	Curtis Breast	Ductal Breast Carcinoma in Situ vs. Normal	ILMN_1694483	-1.05	0.014	-2.4
		Medullary Breast Carcinoma vs. Normal	ILMN_1694483	-1.03	0.03	-1.91
		Breast Carcinoma vs. Normal	ILMN_1694483	-1.04	0.03	-1.85
		Invasive Ductal and Invasive Lobular Breast Carcinoma vs. Normal	ILMN_1694483	-1.02	0.05	-1.58

Supplementary Table 5. Underexpression of GPR50 in Breast cancers (ER positive), Related to Figure 6
Analysis of cancer vs normal tissue with the Oncomine data base considering underexpression of GPR50 in ER-positive breast cancer. Several cancer subtypes with significant P values observed.

Tumor Site	Oncomine Reference	Comparison	Array reporter	Fold change	P value	T test value
Breast	TCGA Breast	Invasive Ductal Breast Carcinoma vs. Normal	A_23_P159721	1.1	9.00E-4	3.24
Breast	Richardson Breast 2	Ductal Breast Carcinoma vs. Normal	208311_at	1.2	0.02	2.3

Supplementary Table 6. Overexpression of GPR50 in Breast cancers (ER positive), Related to Figure 6
Analysis of cancer vs normal tissue with the Oncomine data base considering overexpression of GPR50 in ER-positive breast cancer.
